# Oral health disparities among children with special healthcare needs: a comparative cross-sectional study

**DOI:** 10.1007/s40368-025-01139-x

**Published:** 2025-11-14

**Authors:** R. A. Rajeevan, K. Aparna, M. G. Elenjickal, T. G. Valliaveettil, E. Joseph, J. John, R. G. Varghese, R. Kunnaiah, S. Naik, S. Vellappally, A. A. Abdulah Al kheraif, A. K. John, N. G. Thomas, W. Saleh Al Harbi, G. Schmalz, A. Chopra

**Affiliations:** 1 Department of Periodontology, Pushpagiri College of Dental Sciences, Thiruvalla, Kerala India; 2 Pediatric and Preventive Dentistry, Pushpagiri College of Dental Sciences, Thiruvalla, Kerala India; 3 Pratheeksha Child Development Centre, Pushpagiri Institute of Medical Sciences and Research Center, Thiruvalla, Kerala India; 4 Department of Community Medicine, Pushpagiri Institute of Medical Sciences and Research Centre, Thiruvalla, Kerala India; 5 MES Dental College and Hospital, Malaparamba, Perinthalmanna, Malappuram, Kerala India; 6 Development and Innovation in Oral and Dental Health Research Chair, Department of Dental Health, College of Applied Medical Sciences, King Saud University, Riyadh, Saudi Arabia; 7 Department of Dental Health, King Saud University, Riyadh, Saudi Arabia; 8 Department of Conservative Dentistry and Periodontology, Medizinische Hochschule Brandenburg (MHB) Theodor Fontane, Brandenburg an der Havel, Germany; 9 Department of Periodontology, Oral Medicine and Oral Surgery, Charité–University Medicine Berlin, Corporate member of Freie Universität Berlin, Humboldt–Universität zu Berlin, and Berlin Institute of Health, Berlin, Germany; 10 Stem Cell and Tissue Engineering Laboratory, Pushpagiri Institute of Medical Sciences and Research Centre, Thiruvalla, Kerala India

**Keywords:** Neurodevelopmental disorders, Special healthcare needs, Oral health disparities, Diagnosis-specific interventions, Pediatric dentistry

## Abstract

**Purpose:**

Children with special healthcare needs (SHCN) often experience poorer oral health due to limitations in self-care, behavioral challenges, medical conditions, and reduced access to specialized dental services. This study aimed to assess and compare oral health behaviors, risk factors, and caries experience between children with SHCN and healthy controls, and to identify predictors of poor oral health outcomes.

**Method:**

In this cross-sectional study, 300 children (150 SHCN, 150 controls) underwent clinical examinations. Caries experience was assessed using the decayed, missing, and filled teeth (dmft/DMFT) indices, while oral hygiene status was evaluated with the Plaque Index and Gingival Index. Occlusal characteristics, dental trauma, and oral habits were also recorded. Behavioral assessment was performed using the Frankl Behavior Rating Scale (FBRS).

**Results:**

Children with SHCN demonstrated higher resistance to toothbrushing (42.7% vs. 6.7%, p = 0.001), more frequent swallowing of toothpaste (32.0% vs. 0%, p = 0.001), and a greater prevalence of habits such as mouth breathing (30.7% vs. 8.7%, p = 0.001). Interestingly, the control group showed significantly higher mean dmft scores (5.07 ± 3.56) compared to SHCN children (2.64 ± 4.22, p = 0.001). Regression analysis identified group (B = 2.44, p < 0.001) and age (B = − 0.125, p = 0.002) as significant predictors, explaining 12.2% of the variance. SHCN children exhibited distinct behavioral risk factors, while younger age was associated with higher caries experience.

**Conclusion:**

Children with SHCN exhibited significantly poorer oral health behaviors, including resistance to brushing, swallowing toothpaste, and abnormal oral habits, compared to controls. Interestingly, despite these behavioral disadvantages, caries experience was higher in the control group, underscoring the multifactorial and complex nature of caries development. Group type and age emerged as significant predictors of dmft.

## Introduction

Children and adolescents with SHCN are defined as having any physical, developmental, mental, sensory, behavioural, cognitive, or emotional impairment or limiting condition that requires medical management, healthcare intervention, and/or specialized services. Such conditions may be congenital, developmental, or acquired through disease, trauma, or environmental causes. They often impose restrictions on daily self-maintenance activities or significantly limit major life functions. Healthcare for this population requires specialized knowledge, heightened awareness, and adaptive measures that go beyond routine care (Bethell et al. 2002).

Providing optimal oral healthcare for SHCN children is essential to improve their overall health and quality of life (Alamri 2022). However, these children face considerable barriers to accessing dental services, resulting in higher rates of untreated disease and poorer oral health outcomes than their typically developing peers. With increasing age, their oral health often worsens due to the combined effects of medical conditions, behavioural difficulties, and socioeconomic constraints. Common challenges include poor cooperation during dental procedures, reduced preventive care, and overburdened caregivers who may prioritize medical concerns over daily oral hygiene. Research gaps exist in understanding caries prevalence, molar–incisor hypomineralization, orthodontic problems, traumatic dental injuries, and preventive behaviours in this population (Adeghe et al. 2024; Patidar et al. 2022).

Comprehensive evaluation of oral health across different disabilities can provide valuable insights for designing tailored preventive and therapeutic strategies (Xiang et al. 2020). Incorporating oral health screenings into routine rehabilitative and medical care can facilitate early diagnosis and management, bridging the divide between dental and medical services (George et al. 2024). Current research shows deficiencies: few comparative studies across disability types (Anders and Davis 2010; Wilson et al. 2019), limited standardized assessment tools (Kralikova et al. 2009), and underdeveloped preventive strategies. Barriers are further amplified by inadequate caregiver support (Xiang et al. 2020), scarcity of trained dental providers, and poor medical–dental integration (Stein Duker et al. 2020). In India, children with SHCN are particularly vulnerable due to limited access, low awareness, and socioeconomic obstacles (Pandiyan et al. 2023; Vishnu et al. 2021).

This study systematically addresses these gaps by evaluating oral health status, preventive behaviours, and unmet treatment needs among SHCN children. The findings will guide disability-specific preventive protocols, caregiver education, and interdisciplinary care models, while generating comparative data and proposing standardized screening tools. Ultimately, this work aims to support the development of concepts to reduce oral health inequities and improve the quality of life for this vulnerable group by identifying significant predictors influencing oral health outcomes, which can serve as approaches for future research.

## Materials and methods

### Study design and setting

This cross-sectional study was conducted between November 2023 and November 2024 at the Pratheeksha Child Development Centre, Pushpagiri Institute of Medical Sciences, and the Department of Periodontology, Paediatric and Preventive Dentistry, Pushpagiri College of Dental Sciences, Kerala, India. The study protocol was approved by the Institutional Ethics Committee (Ref. No. PIMS/IEC/2023/45), and all procedures were performed in accordance with the Declaration of Helsinki.

### Study population and sampling

A total of 300 children aged 3–12 years were enrolled. Participants were recruited via convenience sampling. All eligible SHCN children attending the outpatient and therapy departments during the study period were invited consecutively until the target sample size for that group was reached. To ensure comparability, controls were purposively selected from a larger pool of school-going and outpatient children from the same institution to achieve overall matching for age (± 6 months) and gender. A few SHCN children could not participate due to non-cooperation or caregiver unavailability despite repeated behaviour management attempts.

Participants were divided into two groups: the SHCN group (n = 150) and a matched healthy control group (n = 150). SHCN participants were recruited from the outpatient and therapy departments of the Pratheeksha Child Development Centre, while controls were recruited from local schools and pediatric outpatient clinics of the same institution. Controls were matched to SHCN participants for age (± 6 months) and gender to ensure comparability. Due to the heterogeneity of the SHCN population, exact matching was not feasible, and some variability in demographic characteristics remained.

The SHCN group included children with at least one diagnosed condition, such as neurodevelopmental disorders (e.g., autism), intellectual disability, speech and language disorders, motor disorders (e.g., cerebral palsy), developmental delay, seizure disorders, or multiple/complex disabilities (e.g., Down syndrome). Categories were defined based on the Diagnostic and Statistical Manual of Mental Disorders (DSM-5) and International Classification of Diseases (ICD-11) diagnostic criteria. Diagnoses were confirmed through official medical records and recent consultation notes from pediatricians, neurologists, psychiatrists, or speech–language pathologists. Caregiver interviews further verified diagnosis, treatment history, and duration of the condition. Children with more than one condition were classified under multiple/complex disabilities. Children with no diagnosed medical, neurological, or developmental conditions formed the control group.

### Inclusion and exclusion criteria

Eligible participants were children aged 3–12 years whose parents or legal guardians provided written informed consent. SHCN children required a documented diagnosis confirmed by a pediatrician or relevant specialist. Exclusion criteria included acute systemic illness at the time of examination, incomplete clinical or behavioral data, or persistent non-cooperation despite standard behaviour management techniques. These criteria ensured participant safety and reliable assessments, and multiple behaviour management attempts were made before exclusion.

### Sample size calculation

Sample size was calculated using G*Power version 3.1 for a chi-square test comparing proportions between SHCN and control groups. Assuming an effect size (w) of 0.3 (moderate association per Cohen’s convention), a power of 95% (1–β = 0.95), and α = 0.05, the required sample size was approximately 134 per group. The final sample size of 300 (150 SHCN and 150 controls) was determined after considering both statistical and practical factors. This increase accounted for possible dropouts, incomplete data, and non-cooperation during examinations. Given the heterogeneity of the SHCN population, a larger sample enabled subgroup analyses and improved the precision and generalizability of results, while remaining feasible within the study sites and recruitment period.

### Data collection

Data were collected by calibrated examiners through structured interviews and clinical examinations.

### Demographic and background data

Parents or caregivers completed a structured questionnaire covering: age, gender, medical history, birth information, oral hygiene practices (brushing frequency, fluoride toothpaste use, parental assistance), dietary preferences (cariogenic vs. non-cariogenic, based on a food frequency chart) (Bowen et al. 2012), and reported oral habits (mouth breathing, bruxism, nail biting, object biting, self-injurious behaviors).

### Behavioral and functional assessments

Behavioral cooperation was evaluated using the FBRS (Frankl 1962), a validated four-point ordinal scale: (1) Definitely Negative, (2) Negative, (3) Positive, (4) Definitely Positive. Ratings were assigned by the examiner based on overall behavior. The FBRS informed adaptations in the examination protocol (e.g., knee-to-knee positioning, distraction techniques) to optimize comfort and data completeness.

### Clinical examination

Oral examinations were conducted in a child-friendly environment using either an adjustable dental chair or a knee-to-knee position, depending on age, cooperation, and physical limitations. Sterile diagnostic instruments (mouth mirror, explorer, periodontal probe) and headlamps/flashlights were used for adequate illumination. The Tell-Show-Do technique was consistently applied to encourage cooperation.

### Oral health assessments

Standardized clinical examinations assessed:

1. *Oral hygiene and gingival health* determined via Plaque Index (Silness and Löe 1964) and Gingival Index (Löe and Silness 1963).
2. *Caries experience* recorded via dmft/DMFT indices (WHO 2013), with lesion activity classified using Nyvad’s criteria (WHO 2013).
3. *Oral habits* identified via clinical observation and caregiver reports, documenting parafunctional behaviors (mouth breathing, digit sucking, bruxism, lip/nail biting, self-injury).
4. *Dental trauma* classified using Andreasen’s criteria (enamel fracture, luxation, avulsion).

### Examiner calibration

All clinical examinations were performed by two postgraduate residents Periodontology and Pediatric and Preventive Dentistry who had prior experience in oral health assessment of children with SHCN. Before data collection, both examiners underwent a comprehensive training and calibration session led by a senior faculty member Department of Periodontology and Pediatric and Preventive Dentistry, Pushpagiri College of Dental Sciences to ensure uniform understanding and application of diagnostic criteria.

Calibration involved the clinical assessment of 20 children (not included in the study sample), followed by discussion and consensus on diagnostic discrepancies. Reliability was re-evaluated after one week on 10% of the study participants to determine intra- and inter-examiner agreement. The Cohen’s kappa values for categorical variables and Intraclass Correlation Coefficients (ICC) for quantitative measures were both above 0.85, indicating excellent reproducibility.

### Statistical analysis

Data were analyzed using SPSS version 27 (IBM Corp., Armonk, NY, USA). Descriptive statistics (means, standard deviations, frequencies) summarized demographic and clinical data. Group differences were assessed with inferential statistics. Multivariable linear regression was used to identify predictors of dmft scores in SHCN children. Independent variables included group type, age, brushing frequency, resistance to brushing, and preference for sweets. The analysis assessed predictor significance and the variance in dmft scores explained by the model.

## Results

A total of 300 children were enrolled, comprising 150 with SHCN and 150 without SHCN as a comparison group. Gender distribution was similar between groups (SHCN: 56% male; Controls: 49% male; p = 0.248). All neurodevelopmental conditions, including intellectual disability (19.3%), speech and language disorders (10.7%), motor disorders (14.7%), and multiple/complex disabilities (18%), were reported exclusively in the SHCN group, with none observed among controls (p < 0.001). The mean age of participants was comparable between groups (SHCN: 8.4 ± 8.2 years; Controls: 8.1 ± 2.5 years; p = 0.674) (Table 1).

**Table 1:** Demographic and diagnostic characteristics of the study population (SHCN vs. control)

Variable		SHCN n (%)	Control group n (%)	p-value
Gender	Male	84 (56)	74 (49)	χ^2^ = 1.337, p = 0.248
	Female	66 (44)	76 (51)	
Neurodevelopmental disorder		34 (22.7)	0 (0)	χ^2^ = 300.00, p < 0.001*
Intellectual disability		29 (19.3)	0 (0)	
Speech and language disorder		16 (10.7)	0 (0)	
Motor disorder		22 (14.7)	0 (0)	
Developmental delay		14 (9.3)	0 (0)	
Seizure disorder		8 (5.3)	0 (0)	
Multiple/complex disabilities		27 (18)	0 (0)	
Nil		0 (0)	150 (100)	
Age (Mean ± SD)		8.4 ± 8.2	8.1 ± 2.5	t = 0.422, p = 0.674

Data are presented as mean ± standard deviation (SD) or percentage (%)

*p < 0.05 is considered statistically significant

### Preventive oral health behaviors

Comparison of preventive oral health behaviors revealed significant differences between the SHCN and control groups (Table 2). SHCN children were significantly more likely to brush less than once daily (5.3% vs. 0%, p = 0.012), use non-standard brushing aids (finger: 10.7%; toothbrush without toothpaste: 25.3%; p < 0.001), and require assisted brushing (53.3% vs. 0%, p < 0.001). Resistance to toothbrushing was common in SHCN children (42.7%) but rare in controls (6.7%, p < 0.001). Additionally, SHCN children more frequently disliked toothbrushes (28.7% vs. 3.3%) and toothpaste (26% vs. 1.3%), and one-third habitually swallowed toothpaste (32% vs. 0%, p < 0.001).

**Table 2:** Oral health behaviors and practices among the study population (SHCN vs. control)

Oral Health Behavior	SHCN n (%)	Control group n (%)	Chi-square value	p-value
Brushing frequency				
< 1 time/day	8 (5.3)	0 (0)	48.56	0.012*
1 time/day	103 (68.7)	112 (74.7)		
> 1 time/day	39 (26)	38 (25.3)		
Uses a brushing aid				
Toothbrush only	38 (25.3)	0 (0)	68.85	0.001*
Toothbrush + Toothpaste	94 (62.7)	150 (100)		
Finger	16 (10.7)	0 (0)		
Powered toothbrush	2 (1.3)	0 (0)		
Brushing method				
Self	45 (30)	88 (58.7)	109.64	0.001*
Assisted	80 (53.3)	0 (0)		
Self + Assisted	25 (16.7)	62 (41.3)		
Resists brushing				
No	86 (57.3)	140 (93.3)	52.31	0.001*
Yes	64 (42.7)	10 (6.7)		
Does not like toothbrush				
No	107 (71.3)	145 (96.7)	35.81	0.001*
Yes	43 (28.7)	5 (3.3)		
Does not like toothpaste				
No	111 (74)	148 (98.7)	38.68	0.001*
Yes	39 (26)	2 (1.3)		
Swallows’ toothpaste				
No	102 (68)	150 (100)	57.14	0.001*
Yes	48 (32)	0 (0)		
Toothbrushing method (Finger/Brush/Both)				
Not aware	2 (1.3)	0 (0)	123.46	0.001*
Only finger brushing	34 (22.7)	78 (52)		
Only toothbrush use	92 (61.3)	4 (2.7)		
Both finger & toothbrush	22 (14.7)	68 (45.3)		

Data are presented as a percentage (%)

*p < 0.05 is considered statistically significant

### Dietary behaviors

Dietary analysis revealed contrasting patterns (Table 3). A preference for sweets was higher among controls (96%) than among SHCN children (59.3%, p < 0.001), and control participants consumed sweets more frequently. Conversely, SHCN children were more likely to receive sweets as rewards (16.9% vs. 4.0%, p < 0.001). Preference for soft-textured foods was greater among controls (46% vs. 33.3%, p = 0.025). Rinsing after meals was significantly more common in SHCN children (80% vs. 40%, p < 0.001). No significant differences were observed between the groups regarding fussy eating (p = 0.803) or food pouching behaviors (p = 0.441).

**Table 3:** Caries experience assessed by dmft/DMFT indices in the study population (SHCN vs. control)

Behaviour	SHCN n (%)	Control group n (%)	Chi-square value	p-value
Preference for sweets				
No	61 (40.7)	6 (4.0)	123.46	0.001*
Yes	89 (59.3)	144 (96)		
Frequency – Very often				
Rarely	64 (42.7)	0 (0)	85.06	0.001*
Once in a while	43 (28.7)	95 (63.3)		
Very often	43 (28.7)	55 (36.7)		
Sweets as a reward				
No	123 (83.1)	144 (96)	13.28	0.001*
Yes	25 (16.9)	6 (4)		
Fussy eating				
No	142 (94.7)	141 (94)	0.06	0.803
Yes	8 (5.3)	9 (6)		
Prefers soft texture foods				
No	100 (66.7)	81 (54)	5.03	0.025*
Yes	50 (33.3)	69 (46)		
Pouches food in the mouth				
No	137 (91.3)	133 (88.7)	0.59	0.441
Yes	13 (8.7)	17 (11.3)		
Rinses after food				
No	30 (20)	90 (60)	50.00	0.001*
Yes	120 (80)	60 (40)		

Data are presented as mean ± SD

*p < 0.05 is considered statistically significant

### Abnormal oral habits

Parafunctional behaviors were disproportionately represented in the SHCN group (Table 4). Mouth breathing (30.7% vs. 8.7%, p < 0.001), thumb sucking (2.7% vs. 0%, p = 0.044), and bruxism (8% vs. 0%, p = 0.001) were more prevalent in SHCN children. Lip biting occurred only in controls (2.7%, p = 0.044), while tongue biting and self-injurious behaviors were not observed in either group and were therefore not significantly different.

**Table 4:** Abnormal oral habits in the study population (SHCN vs. control)

Oral Habit	SHCN n (%)	Control group n (%)	Chi-square value	p-value
Mouth breathing				
No	104(69.3)	137 (91.3)	χ^2^ = 22.98	0.001*
Yes	46 (30.7)	13 (8.7)		
Thumb sucking				
No	146 (97.3)	150 (100)	χ^2^ = 4.05	0.044*
Yes	4 (2.7)	0 (0)		
Bruxism				
No	138 (92)	150 (100)	χ^2^ = 12.50	0.001*
Yes	12 (8)	0 (0)		
Lip biting				
No	150 (100)	146 (97.3)	χ^2^ = 4.05	0.044*
Yes	0 (0)	4 (2.7)		
Tongue biting				
No	148 (98.7)	150 (100)	χ^2^ = 2.01	0.156
Yes	2 (1.3)	0 (0)		
Self-injurious behavior				
No	150 (100)	150 (100)	–	–
Yes	0 (0)	0 (0)		

Data are presented as a percentage (%)

*p < 0.05 is considered statistically significant

### Clinical oral health indices

**Fig. 1:**
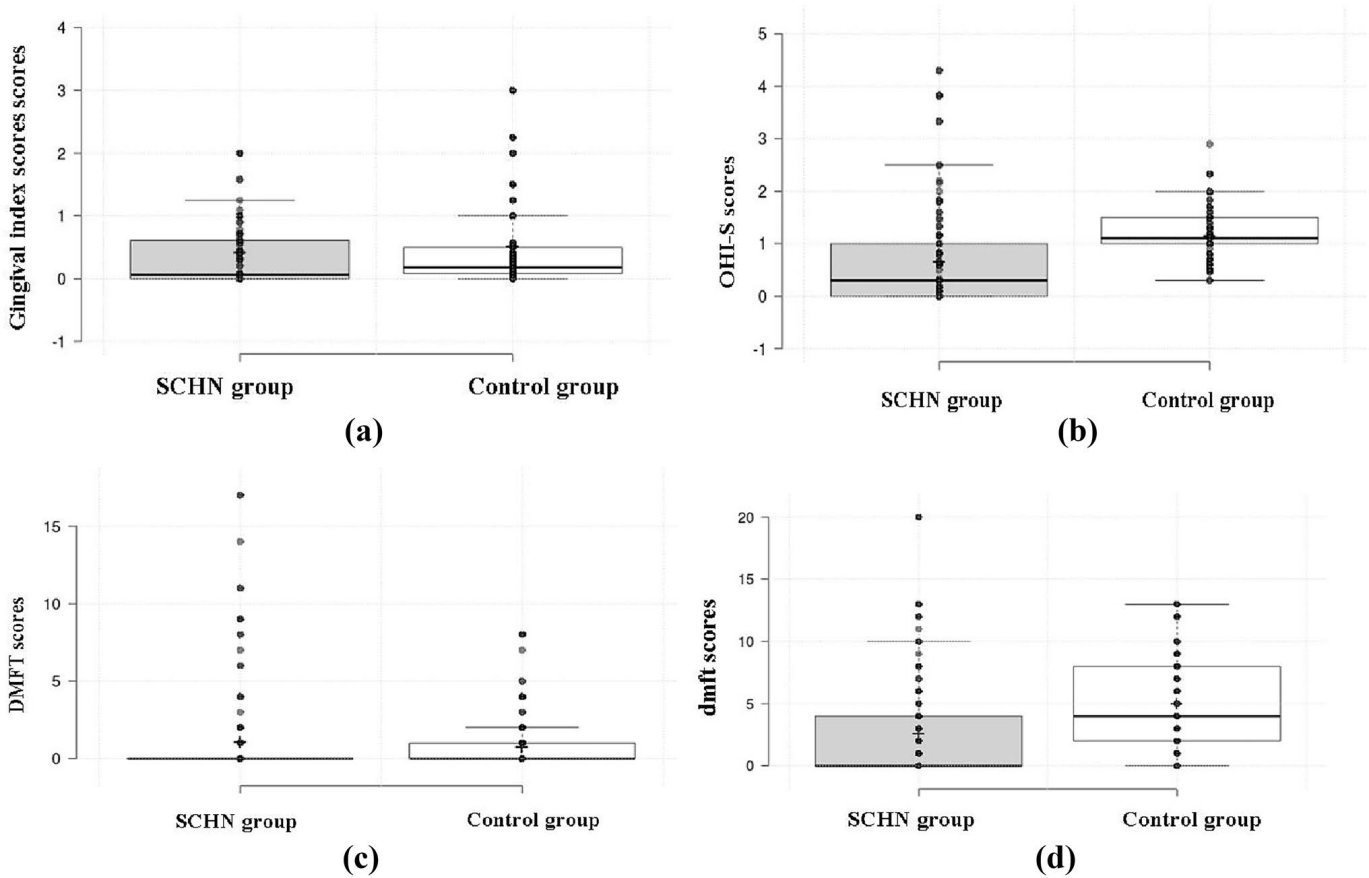
Boxplots of oral health indices in children with SHCN and healthy controls: (**a**) Modified Gingival Index (MGI), (**b**) Debris Index (OHI-S), (**c**) DMFT, and (**d**) dmft. Lines represent medians, boxes interquartile ranges, and whiskers minimum–maximum values. Controls showed significantly higher OHI-S and dmft scores (p < 0.001; mean dmft 5.07 vs. 2.64). SHCN participants had lower OHI-S scores (0.70 vs. 1.16) but greater variability. DMFT means were similar, although SHCN participants displayed more outliers. Gingival Index was slightly higher in controls (0.52 vs. 0.43)

Comparisons of gingival and oral hygiene parameters are summarized in Table 5 and Fig. 1. Modified Gingival Index (MGI) scores were slightly higher in controls (0.52 ± 0.75) than in SHCN children (0.43 ± 0.61), but the difference was not significant (p = 0.238). The Plaque Index (OHI-S) was significantly higher in controls (1.16 ± 0.41 vs. 0.69 ± 0.92; p < 0.001). While DMFT scores were comparable (SHCN: 1.07 ± 3.08; Controls: 0.77 ± 1.66; p = 0.311), the mean dmft score was higher in controls (5.07 ± 3.56) than in SHCN children (2.64 ± 4.22; p < 0.001), indicating a greater caries burden in the primary dentition of controls.

**Table 5:** Gingival and oral hygiene parameters of the study population (SHCN vs. control)

Index	SHCN (Mean ± SD)	Control group (Mean ± SD)	t value	p-value
Modified Gingival Index (MGI)	0.43 ± 0.61	0.52 ± 0.75	− 1.18	0.238
Plaque Index (OHI-S)	0.69 ± 0.92	1.16 ± 0.41	− 5.72	0.001*
DMFT score	1.07 ± 3.08	0.77 ± 1.66	1.02	0.311
dmft score	2.64 ± 4.22	5.07 ± 3.56	− 5.37	0.001*

Data are presented as mean ± SD

*p < 0.05 is considered statistically significant

Boxplots (Fig. 1) illustrate group differences in oral health parameters. In OHI-S scores (Fig. 1a), SHCN participants showed lower mean values (0.70 vs. 1.16) but greater variability compared to the comparison group, reflecting the heterogeneous nature of disabilities within this population. DMFT scores (Fig. 1b) were comparable between groups, though higher outliers were observed in the SHCN group. For dmft scores (Fig. 1c), the comparison group exhibited a higher mean (5.07 vs. 2.64) with wide distributions in both groups. Gingival Index scores (Fig. 1d) were marginally higher in the comparison group (0.52 vs. 0.43). Overall, the greater variability observed in OHI-S and DMFT scores among SHCN participants likely reflects intra-group differences in disability type, functional limitations, and oral care practices, whereas the comparison group showed a higher central tendency for dmft. These findings highlight both disability-specific effects and the influence of heterogeneity on score dispersion.

### Predictors of caries experience

A multivariable linear regression was performed to identify predictors of dmft scores (Table 6). The model was statistically significant (F(5, 291) = 8.092, p < 0.001), explaining 12.2% of the variance (adjusted R^2^ = 0.107). Group type (B = 2.44, p < 0.001) and age (B = − 0.125, p = 0.002) emerged as significant predictors: SHCN status was associated with higher dmft values, whereas increasing age was associated with lower dmft. Brushing frequency, resistance to brushing, and sweet preference were not significant predictors (Table 6).

**Table 6:** Multivariable linear regression analysis for predictors of dmft scores in the study population

Predictor	B	SE	t	p-value	95% CI (B)
(Constant)	1.323	1.132	1.168	0.244	− 0.906 to 3.552
Group (SCNC = 1, Control = 2)	2.440	0.549	4.443	< 0.001	1.359 to 3.521
Age (in years)	− 0.125	0.039	− 3.185	0.002	− 0.203 to − 0.048
Toothbrushing Frequency (ordinal)	0.224	0.473	0.474	0.636	− 0.707 to 1.154
Resistance During Brushing (Yes = 1)	− 0.255	0.607	− 0.420	0.675	− 1.449 to 0.940
Preference for Sweets (Yes = 1)	− 0.401	0.604	− 0.664	0.507	− 1.589 to 0.788

R^2^ = 0.122; Adjusted R^2^ = 0.107; F(5, 291) = 8.092, p < 0.001

Regression coefficients (B), standard error (SE), and significance (p-value) are presented

*p < 0.05 is considered statistically significant

## Discussion

This study provides a comprehensive comparison of oral health, habits, and risk factors between children with SHCN and healthy controls. The findings are consistent with much of the existing literature while also revealing unique differences that enhance understanding of oral health disparities in this vulnerable group (Bradley and McAlister 2004; Oredugba and Akindayomi 2008; Purohit et al. 2010).

The study results confirmed associations reported previously, including behavioral challenges and oral habits linked to SHCN, while highlighting that caregiver involvement may mitigate some risks. For example, plaque scores in certain SHCN subgroups were comparable to controls, suggesting that active parental supervision plays a protective role, which is consistent with earlier reports showing reduced disease severity when caregivers support daily hygiene routines (Alkhabuli et al. 2019; Gadiyar et al. 2018).

Children with autism, intellectual disabilities, Down syndrome, and cerebral palsy have consistently been reported to exhibit higher risks of malocclusion, gingivitis, and caries due to behavioral resistance, sensory sensitivities, motor impairments, medication side effects, and caregiver dependency (Sabbarwal et al. 2018). Surprisingly, our findings showed higher mean dmft scores in controls than in SHCN children, contradicting much of the literature (Bradley and McAlister 2004; Patidar et al. 2022). However, this is not unprecedented; some studies report equal or lower caries rates in SHCN populations, potentially due to dietary restrictions, breastfeeding patterns, or intensified parental care (Oredugba and Akindayomi 2008). Regression analysis confirmed group status and age as significant predictors: SHCN status was associated with lower caries, while younger age was linked to higher dmft, consistent with the natural exfoliation of primary teeth.

The zero percent observed for assisted brushing in the comparison group is likely an underestimate, as preschool-aged children normally require some parental assistance with tooth brushing. This discrepancy may reflect caregiver reporting bias, the age distribution of the comparison group, or underestimation during data collection. Therefore, these results should be interpreted with caution.

DMFT values were comparable between groups, suggesting similar caries experience in permanent dentition. This could indicate that protective parental involvement in younger years diminishes over time, or that risk factors converge as children grow older. Longitudinal research is needed to confirm these trajectories.

Dietary habits added further complexity. While controls showed significantly greater sweet preference and higher consumption frequency, SHCN children more often received sweets as rewards. Interestingly, rinsing after meals was far more common among SHCN children, potentially reflecting caregiver-led interventions. These compensatory practices may explain the unexpectedly lower caries burden in the SHCN group, echoing earlier evidence on the protective impact of structured routines (Alkhabuli et al. 2019; Purohit et al. 2010).

Oral parafunctional habits were significantly more prevalent in SHCN, including mouth breathing, thumb sucking, and bruxism. Such habits are well-documented in neurodevelopmental disorders and contribute to gingival inflammation, altered craniofacial growth, malocclusion, tooth wear, and myofascial pain (Alkhabuli et al. 2019; Bradley and McAlister 2004). While not direct predictors of dmft in this study, their contribution to long-term oral morbidity warrants early preventive intervention.

Predictive modeling underscored SHCN status and younger age as independent predictors of caries in the primary dentition. However, the relatively low R^2^ value reflects the multifactorial nature of caries and highlights unmeasured influences such as socioeconomic status, parental education, fluoride history, and access to dental care (Frank et al. 2019). The lack of significance for behaviors such as brushing frequency or sweet preference in the multivariate model reinforces the notion that broader contextual factors, such as community-level fluoride exposure and structural barriers to healthcare, may outweigh individual-level practices (Pandiyan et al. 2023).

Access to dental care remains a critical determinant. Financial barriers, transportation challenges, provider inexperience, and behavioral management difficulties are frequently cited obstacles for SHCN families. As prior studies confirm, empowering caregivers and adequately training providers can significantly improve preventive practices and service utilization, pointing toward structural and educational interventions as essential strategies (Ningrum et al. 2021; Puthiyapurayil et al. 2022).

Condition-specific patterns also emerged: children with Down syndrome often present with class III malocclusion and enamel defects despite good hygiene (Sabbarwal et al. 2018); cerebral palsy increases periodontal risk through impaired motor control (Puthiyapurayil et al. 2022); epilepsy introduces drug-related risks such as gingival enlargement (Patidar et al. 2022); while autism and intellectual disabilities demand individualized behavioral strategies. Conversely, cleft conditions require multidisciplinary planning rather than enhanced caries prevention. These differences affirm the need for tailored rather than generalized oral health strategies in SHCN populations.

Systematic reviews further emphasize that oral health disparities in SHCN result from complex interactions among biological, behavioral, and systemic factors (Ningrum et al. 2021). Protective elements such as structured routines, preventive visits, and caregiver engagement can mitigate risk, even in high-risk groups (Oredugba and Akindayomi 2008; Purohit et al. 2010). The current findings add to this evidence, underscoring that interventions must address both the immediate clinical needs and the broader systemic and social determinants.

Limitations of this study include reliance on caregiver-reported data, which may introduce recall bias; heterogeneity within the SHCN cohort, which could mask condition-specific differences; and the absence of confounders such as socioeconomic indicators and detailed fluoride exposure. These factors limit the generalizability and completeness of the conclusions. The SHCN group included children with diverse medical and developmental conditions, which may introduce variability in oral health and behavioral profiles. However, this heterogeneity was intentionally maintained to reflect real-world pediatric dental populations and improve generalizability. Future studies focusing on specific diagnostic categories are warranted to further clarify condition-specific patterns.

The exclusion criteria ensured safety and data quality but may have underrepresented the most severe SHCN cases, making the findings conservative. Future studies focusing on severe SHCN subgroups with adapted assessment protocols are recommended. The cross-sectional design limits causal inference, and monocentric recruitment may reduce generalizability. The cohort was heterogeneous, and future studies may consider sample size adjustments in the context of comparable literature. Finally, to assess clinical implications, prospective studies informed by these cross-sectional results are needed.

## Conclusion

Children with SHCN show distinct oral habits, dietary patterns, and parafunctional behaviors compared to peers. Higher dmft scores and behavioral challenges influence oral health, while group status and age emerged as significant predictors of caries. Surprisingly, lower plaque scores suggest active caregiver involvement despite poorer hygiene practices. These findings highlight the complex interplay of disability, age, and parental engagement, emphasizing the need for targeted preventive and therapeutic strategies. Tailored special care approaches can improve oral health outcomes and challenge the assumption that SHCN children inherently experience poorer oral health.

## Data Availability

The data supporting the findings of this study are available from the corresponding author upon reasonable request.
